# Digital Approaches to Pain Assessment Across Older Adults: A Scoping Review

**DOI:** 10.3390/healthcare14020149

**Published:** 2026-01-07

**Authors:** Leanne McGaffin, Gary Mitchell, Tara Anderson, Arnelle Gillis, Stephanie Craig

**Affiliations:** School of Nursing and Midwifery, Queen’s University Belfast, Belfast BT9 7BL, UK; lmcgaffin01@qub.ac.uk (L.M.); gary.mitchell@qub.ac.uk (G.M.); tanderson@qub.ac.uk (T.A.); agillis03@qub.ac.uk (A.G.)

**Keywords:** digital pain assessment, facial recognition, PainChek, ePAT, older adults, dementia, nursing, scoping review

## Abstract

Background: Effectively managing pain in adults remains challenging, particularly in individuals with cognitive impairment or communication difficulties. Digital technologies, including artificial intelligence (AI)-enabled facial recognition and mobile applications, are emerging as innovative tools to improve the objectivity and consistency of pain evaluation. This scoping review aimed to map the current evidence on digital pain-assessment tools used with adult and older populations, focusing on validity, reliability, usability, and contributions to person-centred care. Methods: The review followed the Joanna Briggs Institute methodology and Arksey and O’Malley framework and was reported in accordance with PRISMA-ScR guidelines. Systematic searches were conducted in PubMed, CINAHL Complete, Medline (ALL), and PsycINFO for English-language studies published from 2010 onwards. Eligible studies included adults (≥18 years) using digital tools for pain assessment. Data extraction and synthesis were performed using Covidence, and findings were analyzed thematically. Results: Of 1160 records screened, ten studies met inclusion criteria. Most research was quantitative and conducted in high-income clinical settings. Five tools were identified: ePAT/PainChek^®^, Painimation, PainCAS, Pain Clinical Assessment System, and Active Appearance Model. Four key themes emerged: (1) Validity and Reliability of Digital Pain Assessment Tools; (2) Comprehensive Pain Evaluation Across Contexts (Rest vs. Movement); (3) Usability and Integration into Clinical Practice; (4) Enabling Person-Centred Pain Management and Future Directions. Conclusions: Emerging evidence suggests that facial-recognition-based digital pain-assessment tools may demonstrate acceptable psychometric performance and usability within dementia care settings in high-income countries. However, evidence relating to broader adult populations, diverse care contexts, and low-resource settings remains limited, highlighting important gaps for future research.

## 1. Introduction

Pain is a complex, multifaceted phenomenon that remains one of the most pervasive and challenging issues in global health [1]. The International Association for the Study of Pain [2] defines pain as “an unpleasant sensory and emotional experience associated with, or resembling that associated with, actual or potential tissue damage,” emphasising its inherently subjective and multidimensional nature. This conceptualisation acknowledges that pain is influenced by biological, psychological, and social factors, and that the inability to communicate pain verbally does not negate its presence [3]. Understanding pain as a personal and contextual experience necessitates a biopsychosocial approach to assessment and management, positioning nurses at the forefront of clinical evaluation and intervention [4].

Globally, the burden of chronic pain is increasing however the need for better transparency and service-level costing to identify gaps and ensure equitable access to pain services across the healthcare system is urgently required. Estimates suggest that around one in five adults worldwide live with chronic pain [2]. A comprehensive meta-analysis across 52 countries reported a mean global pain prevalence of 53.2%, with national estimates ranging from 23.7% to 78.4%, highlighting significant regional variation in the chronic pain burden [5]. Within Europe, the prevalence of chronic pain in adult populations is estimated at roughly 33%, with musculoskeletal conditions representing the predominant cause [6]. In the United Kingdom, chronic pain affects approximately 28 million adults equating to between one-third and one-half of the adult population [7]. The incidence of pain increases markedly with age, and prevalence among residents of long-term care facilities has been reported to exceed 80% [8]. On a global scale, musculoskeletal disorders particularly low-back pain are among the leading causes of disability, affecting more than 1.5 billion people worldwide [9,10,11,12]. Collectively, these figures highlight the profound human, social, and economic impact of pain and its implications for health systems across diverse contexts.

The aetiology of pain is heterogeneous and often poorly understood [13]. Pain may be classified as acute, chronic, or recurrent, with chronic pain defined by the National Institute for Health and Care Excellence (NICE) [14] as pain persisting or recurring for three months or longer. Chronic pain may be further delineated into primary pain, in which the pain itself constitutes a disease entity without a clear underlying pathology, and secondary pain, which arises as a symptom of another condition such as cancer, osteoarthritis, or neuropathy [15]. Acute pain, by contrast, is typically short-term and self-limiting in response to injury or illness and subsides as recovery progresses. Additional classification frameworks distinguish between nociceptive pain, resulting from tissue injury, and neuropathic pain, arising from damage to the somatosensory nervous system [16]. The complexity of these overlapping mechanisms contributes to the challenges faced by clinicians in diagnosis, assessment, and management.

Within this context, nurses occupy a pivotal role in pain assessment and management. The Nursing and Midwifery Council (NMC) [17] outlines in its professional Code of conduct that nurses are required to uphold the highest standards of care, respect patient individuality, and promote health and well-being, principles that inherently encompass the alleviation and prevention of pain. Likewise, NICE [14] emphasises that effective pain management requires a person-centred approach, incorporating shared decision-making and sensitivity to the social, cultural, and ethical dimensions that shape a patient’s experience of pain. Through their ongoing proximity to patients and their holistic perspective, nurses are uniquely positioned to identify pain, interpret non-verbal cues, and advocate for appropriate interventions.

However, numerous barriers impede the accurate assessment of pain within nursing practice [18]. The subjective nature of pain, differences in patient thresholds and expression, and nurses’ own attitudes, workload pressures, and knowledge gaps contribute to significant variability in assessment quality [19]. Practical obstacles, such as time constraints and the limited availability of validated tools, further hinder comprehensive evaluation [20]. A commissioned review of pain assessment and management in Northern Ireland long-term care settings identified inconsistent documentation, variability in the use of PRN (pro re nata) analgesia, and organisational barriers to effective pain management, reinforcing concerns about the under-recognition and undertreatment of pain in institutional care [21]. Regulatory inspection findings in Northern Ireland have similarly highlighted deficiencies in medicines management and care planning within nursing and residential homes.

Pain assessment tools serve as essential instruments in addressing these challenges, offering structured means of evaluating pain intensity, quality, and impact [22]. Self-report scales, such as the Numeric Rating Scale (NRS), remain the gold standard where feasible, providing patients with autonomy and enabling quantifiable comparison [23]. Yet, for patients unable to communicate effectively, such as those with advanced dementia or cognitive impairment observational tools like the Abbey Pain Scale (APS) are widely employed [24]. The APS has demonstrated acceptable reliability and validity in some studies [25], although recent research has questioned its consistency across populations and highlighted the potential for user bias [24].

The limitations of traditional, paper-based tools have prompted increasing interest in digital and artificial intelligence (AI)-enabled pain assessment technologies [26]. Systems such as PainChek^®^ and the Electronic Pain Assessment Tool (ePAT) utilise automated facial recognition and algorithmic scoring to detect micro-expressions associated with pain, offering real-time, objective data to support clinical decision-making [27]. These technologies claim to provide efficient, reproducible, and time-saving assessments that can be integrated into electronic health records, potentially reducing missed diagnoses and improving patient outcomes. Their adoption within UK care homes and international settings reflects a growing movement toward technologically enhanced nursing practice [28].

Despite these developments, empirical evidence regarding the effectiveness, reliability, and clinical impact of digital pain assessment tools remains limited and fragmented. A systematic synthesis of existing literature is therefore warranted to determine their utility in supporting nurses’ assessments and informing pain management strategies. This scoping review aims to map the current evidence base on digital pain assessment tools, evaluate their practical application within nursing contexts, and identify knowledge gaps for future investigation. By exploring the potential of digital technologies to enhance the accuracy and efficiency of pain assessment, this review seeks to contribute to improved clinical outcomes, patient safety, and overall quality of care in the management of pain across diverse healthcare settings.

## 2. Materials and Methods

### 2.1. Study Design

A scoping review design was adopted to map existing evidence on digital pain assessment tools in adults and older adults. This approach enables the exploration of broad research questions and the identification of conceptual and empirical gaps within a field [29]. Scoping reviews are particularly appropriate for synthesising emerging evidence where diverse study designs exist [30]. Unlike systematic reviews, which focus on effectiveness, scoping reviews aim to chart key concepts, methodological approaches, and knowledge gaps.

A scoping review was conducted following the Joanna Briggs Institute (JBI) methodology for scoping reviews [31], which builds upon Arksey and O’Malley’s earlier framework [32]. This review is reported in line with the Preferred Reporting Items for Systematic reviews and Meta-Analyses extension for Scoping Reviews (PRISMA-ScR) checklist [33]. The completed checklist is available in Supplementary File S1. The review protocol was retrospectively registered in November 2025 with the Open Science Framework (OSF) registration DOI: https://osf.io/fbtjm/overview, (accessed on 9 November 2025). Registering the protocol strengthened methodological rigour and minimized risk of reporting bias.

### 2.2. Search Strategy

An initial exploratory search using Google Scholar was undertaken to assess topic feasibility and determine the scope of available evidence. A comprehensive search of peer-reviewed literature was then conducted across four electronic databases: PubMed, CINAHL Complete, Medline (ALL), and PsycINFO. Searches conducted in August 2025. Search strategies were refined in consultation with the research supervisor to enhance precision and coverage. Both free-text keywords and Medical Subject Headings (MeSH) were used, incorporating Boolean operators (AND/OR) to connect search terms. Search terms included combinations of population descriptors (e.g., “older adults,” “geriatric,” “middle-aged”), exposure variables (e.g., “digital pain assessment,” “PainChek,” “Painimation”), and outcome-related terms (e.g., “pain measurement,” “electronic assessment”). The final search strategies are provided in Supplementary File S2.

Search strategies were developed using two core concept blocks: (1) digital or electronic pain-assessment tools; (2) adult and older-adult populations. In PsycINFO, the digital pain-assessment concept included the terms electronic pain assessment, electronic pain measurement, PainChek, and Painimation, combined using the Boolean operator OR. The population concept included a broad range of adult and ageing-related terms, including adult, aged, middle aged, older adults, older people, elderly, geriatric, senior citizens, and frail elderly, also combined using OR. The two concept blocks were then combined using AND. Preliminary testing of broader digital health terms (e.g., e-health, telehealth, mobile health) retrieved a high volume of non-relevant records. To balance sensitivity and specificity and maintain feasibility, the final search strategy prioritised pain-assessment-specific and tool-based terminology.

### 2.3. Eligibility Criteria

Eligibility criteria were developed using the PEO (Population, Exposure, Outcome) framework [34]. This framework facilitated a structured and transparent approach to defining inclusion and exclusion parameters.

- Population: Adults and older adults (≥18 years); studies focusing on paediatric populations were excluded.
- Exposure: Use of digital pain assessment tools or electronic pain measurement technologies. Paper-based or analogue tools were excluded.
- Outcomes: Studies examining the accuracy, reliability, or clinical applicability of digital pain assessment.

The search was restricted to studies published from 2010 onwards to ensure contemporary relevance, as digital and AI-enabled pain-assessment technologies have advanced rapidly over the past decade. Studies were limited to the English language due to constraints in translation resources, and we acknowledge that this may introduce language bias. Empirical peer-reviewed publications were included in order to map current evidence and key concepts directly related to the clinical use, psychometric performance, and implementation of digital pain-assessment tools in adult populations. Conference abstracts and commentaries were excluded due to their limited methodological detail and lack of full peer review. We acknowledge that these restrictions may have limited retrieval, particularly given the emerging and technology-driven nature of this field.

### 2.4. Data Extraction

All records were imported into Covidence (https://www.covidence.org) for automated duplicate removal and to support a structured screening approach. Data from included studies were extracted using a standardised data extraction form within Covidence. Extracted data included author, year, country, study design, population characteristics, intervention type, outcome measures, and key findings. The process was guided by the PRISMA-ScR framework [33] to ensure transparency in selection and reporting. Stage 1 screening was conducted independently and using a blinded approach by two reviewers (LMcG and SC), with 100% of records dual-screened. Conflicts were resolved by a third reviewer (GM). The same process was applied at full-text screening, this ensured consistency and minimized selection bias in accordance with PRISMA-ScR guidance.

### 2.5. Data Analysis

Extracted data were analysed using a narrative synthesis approach, allowing integration of findings across diverse study designs and measures [35]. Studies were grouped by shared characteristics and intervention types, enabling comparison across methodological features, outcomes, and contexts. A thematic analysis, informed by Braun and Clarke’s [36] six-step framework, was employed to identify patterns and key themes emerging from the data. This method supported a systematic exploration of commonalities and divergences across the included studies, ensuring depth of interpretation while maintaining alignment with the scoping review objectives.

### 2.6. Quality Appraisal

Although formal critical appraisal is not mandatory for scoping reviews, quality assessment was undertaken to enhance transparency and credibility [37]. The Joanna Briggs Institute (JBI) critical appraisal tools were used to evaluate methodological quality and potential bias [38]. The JBI quasi-experimental checklist was applied to nine quantitative studies, and the JBI qualitative checklist was used for one qualitative study. Each study was assessed across domains such as clarity of objectives, appropriateness of design, data collection, and validity of analysis. Most studies achieved high methodological quality, with scores ranging from 5 to 9, indicating overall robustness and reliability of the evidence base.

## 3. Results

### 3.1. Search Results

**Figure 1:**
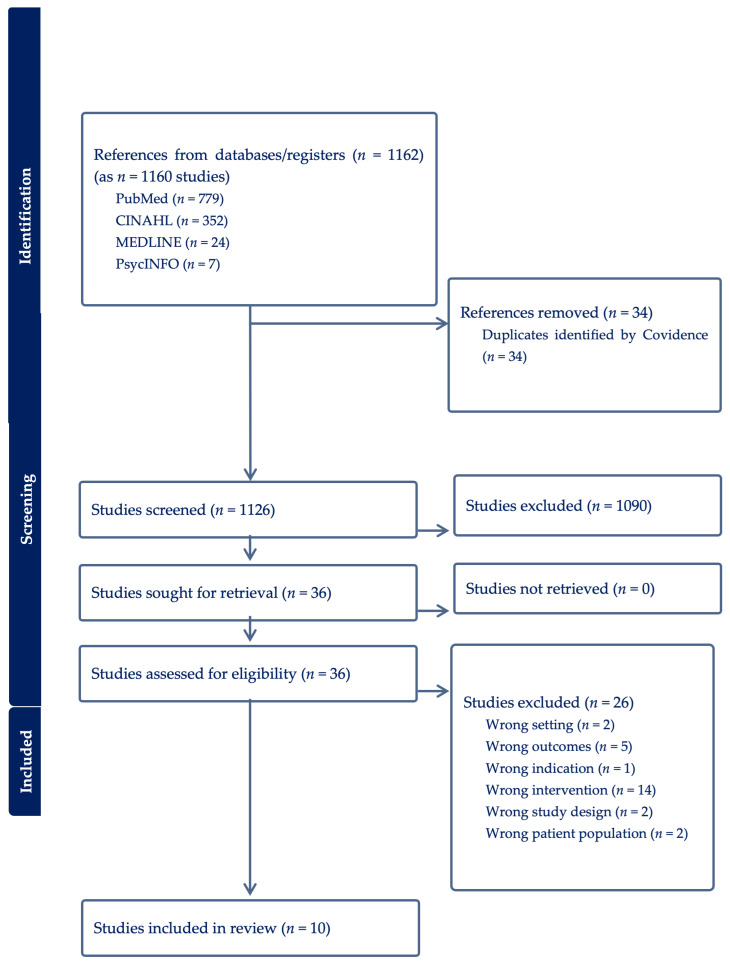
PRISMA-ScR flow diagram.

In the present review, 1160 records were imported, 34 duplicates were excluded, leaving 1126 for screening. Title/abstract screening excluded 1090 studies; 36 full texts were assessed for eligibility. Twenty-six were excluded for reasons including incorrect intervention (*n* = 14), wrong outcome (*n* = 5), wrong population (*n* = 2), irrelevant setting (*n* = 2), wrong study design (*n* = 2) and wrong indication (*n* = 1). Ten studies remained. The study selection process is presented in a PRISMA-ScR flow diagram in Figure 1 [33]. 

### 3.2. Characteristics of Included Studies

Ten studies met the inclusion criteria for this review. A summary of their characteristics is provided in Table 1. The majority (*n* = 9) employed quantitative methodologies [39,40,41,42,43,44,45,46,47], while one adopted a mixed-methods approach. One study [48] utilised a mixed-methods design incorporating both experimental data and patient interviews. None of the included studies employed a purely qualitative approach. The JBI checklist [38] was used to systematically evaluate each design, ensuring consistent assessment of methodological robustness.

### 3.3. Study Setting

All ten studies reported their research settings. Six were conducted in Australia, three in the United States [43], and one in the United Kingdom [47], indicating that digital pain assessment tools remains largely concentrated in higher-income settings. Seven studies were situated in residential or aged-care facilities [39,40,41,42,44,45,47], two in a hospital [46,48], and one in a clinic setting [43].

The predominance of studies conducted in institutional care environments limits the generalisability of findings to community-dwelling populations. Additionally, the concentration of research within two high-income regions (Australia and the United States) may restrict cross-cultural applicability [49].

### 3.4. Sampling

All studies employed purposive (non-probability) sampling, appropriate for the targeted populations of adults and older adults, most commonly aged over 65 years. Across the included papers, sample sizes ranged from 10 to 199 participants, with a mean of approximately 70. The average participant age was around 74 years, with age ranges typically spanning from the mid-60s to the early-80s, reflecting the demographic characteristics of residential and long-term care settings. Gender representation showed a slight predominance of females (averaging around 65% women to 35% men), aligning with the greater proportion of older women in such care contexts. Inclusion criteria were consistently clear, focusing on individuals with dementia or cognitive impairment, and often requiring a minimum duration of residence (e.g., over 3 months). Quantitative included larger cohorts to ensure statistical robustness, while smaller qualitative studies prioritized depth of experience. Overall, the purposive and well-defined sampling strategies enhanced the contextual relevance and transferability of findings across diverse care environments.

### 3.5. Interventions

Five distinct digital pain-assessment tools were identified across the ten studies:

1. Electronic Pain Assessment Tool (ePAT)—used in four studies [39,40,41,42]. ePAT is a point-of-care smartphone application employing facial recognition to analyse micro-expressions across five domains (voice, movement, behaviour, activity, and body). The tool was validated against the Abbey Pain Scale (APS) to assess psychometric and clinometric properties.
2. PainChek—examined in three studies [44,45,47]. This AI-driven evolution of ePAT combines facial analysis with behavioural indicators to produce pain scores. Studies focused on psychometric testing, pain-behaviour mapping, and pain management in dementia care.
3. Pain Clinical Assessment System (PainCAS)—one study [48] evaluated this computer-based self-report tool for chronic pain monitoring and opioid risk documentation.
4. Painimation—one study [43] explored this app enabling patients to describe pain through animated graphics rather than numeric scales.
5. Active Appearance Model (AAM)—one study [46] tested an automated computer vision model detecting pain through coded facial expressions compared to visual analogue scales.

### 3.6. Study Results

A total of four overarching themes were identified from the ten studies included in this scoping review. These were: (1) the comparative validity and reliability of digital pain assessment tools against traditional measures such as the Abbey Pain Scale (APS); (2) the comprehensive evaluation of pain across contexts, including assessments conducted at rest and during movement; (3) the usability and integration of digital platforms within clinical practice; (4) the role of digital assessment tools in enabling person-centred pain management and informing future directions for research and practice. A structured per-study summary of reported quantitative outcomes, including psychometric and related metrics, is provided in Supplementary File S3. Collectively, these themes reflect findings from a small and methodologically heterogeneous body of literature, primarily comprising validation and feasibility studies conducted in high-income, institutional care settings. The evidence base is heavily concentrated in dementia-focused residential aged-care contexts and is dominated by facial-recognition-based tools such as ePAT/PainChek. There is limited representation of community-based settings, non-dementia populations, or acute care environments, and no included studies evaluated implementation or interventional outcomes. As such, the themes should be interpreted as mapping where evidence currently exists, rather than demonstrating comprehensive effectiveness of digital pain assessment across all adult and older-adult populations. Although several studies reported favorable psychometric and usability outcomes, findings varied across tools and contexts, with greater consistency observed in dementia-focused facial-recognition tools than in self-report platforms or non-institutional settings.

#### 3.6.1. Theme 1: Validity and Reliability of Digital Pain Assessment Tools

The comparative validity and reliability of digital pain assessment tools have been central to research examining their effectiveness against established paper-based measures. Across the included studies, there is evidence that digital tools, including ePAT (PainChek), demonstrate fair psychometric performance, aligning closely with traditional methods while introducing elements of increased objectivity through algorithmic and facial recognition technologies. This theme synthesises evidence from Atee et al., Babicova et al., Hoti et al., Jonassaint et al., Pu et al., and Lucey et al. [39,40,41,42,43,44,46,47], focusing on the statistical and conceptual dimensions of validity, reliability, and equivalence in pain assessment.

Atee et al. [39] directly compared the psychometric properties of ePAT with the APS to evaluate concurrent validity and inter-rater reliability. The study demonstrated a positive correlation between APS and ePAT scores, with Pearson’s r = 0.882 (95% CI 0.857–0.903), indicating that both tools generated comparable pain assessments within a narrow time frame. Inter-rater reliability was assessed using Cohen’s kappa, revealing moderate to good agreement between raters. Cronbach’s alpha of 0.925 suggested excellent internal consistency, confirming that ePAT measures the same construct of pain as APS. These findings established ePAT as a valid and reliable comparator to conventional pain scales, with results reproducible across rest and movement contexts.

Building on this, Atee et al. [40] found similarly concurrent validity between ePAT and APS, reporting a Pearson’s correlation coefficient of r = 0.911, indicating high concordance across observation conditions. Internal consistency remained robust, with Cronbach’s alpha values of 0.797 at movement and 0.766 at rest, reinforcing the reliability of the digital tool in detecting pain across different physical states. Atee et al. [41] extended this analysis by examining the performance of the facial recognition domain within ePAT. When evaluated against self-reporting, the face domain achieved good sensitivity in detecting pain, and linear modelling indicated positive agreement between facial recognition outcomes and patient self-reported pain (r = 0.91). Collectively, these findings affirm that digital platforms employing facial recognition are capable of producing reliable and valid assessments comparable to conventional scales while offering enhanced sensitivity to facial indicators of discomfort. Adding nuance to these scale-level correlations, Pu et al. [44] analyzed 1820 assessments from 46 residents and identified six facial expressions with significant positive associations to observational pain (e.g., brow lowering, horizontal mouth stretch, lip parting, nose wrinkling, upper-lip raise, and eye closure). Importantly, they removed the face domain from the composite outcome to avoid circularity, strengthening the inference that these facial cues contribute independent information about pain. This pattern-level evidence supports the construct validity of facial analytics within digital tools and clarifies which facial movements are most informative in dementia care.

The study by Babicova et al. [47] further supported the equivalence between digital and paper-based assessments. Their analysis demonstrated a significant positive correlation between APS and PainChek scores (r = 0.818), reinforcing the psychometric validity of digital assessment tools in clinical populations. Notably, their research was conducted within patients’ usual living environments, enhancing ecological validity and demonstrating that these tools maintain reliability under real-world conditions. This finding indicates comparability between digital and paper-based assessments within this specific care context, particularly where self-report is challenging or unreliable.

Hoti et al. [42] supported the reliability of ePAT through comparative analysis, reporting consistent outcomes across multiple measurement domains. Their study confirmed that mean ePAT scores corresponded closely with APS results both at rest and during movement, demonstrating that the digital platform performs with comparable reliability across varying conditions. The repeated demonstration of positive correlations across these studies collectively supports the premise that digital assessments can replicate the diagnostic accuracy of established paper-based tools.

In addition to quantitative validation, the studies considered how digital platforms might enhance objectivity by reducing human interpretation biases. Atee et al. [39,41] and Babicova et al. [47] highlighted that facial recognition algorithms, which decode facial action units associated with pain, can mitigate the subjectivity inherent in observer-rated or self-reported assessments. By quantifying observable facial expressions, these systems reduce reliance on the assessor’s subjective interpretation and improve consistency across raters. Such automation contributes to standardisation and reproducibility, particularly important when assessing patients with impaired communication abilities, such as those with dementia or cognitive decline. Pu et al.’s [44] action-unit findings are consistent with this argument, indicating that algorithmic detection of selected facial movements can reduce rater subjectivity by focusing assessors on reproducible visual features linked to observed pain behaviors.

Lucey et al. [46] provided additional insight into how facial action unit analysis can objectively capture distress and pain. Their findings demonstrated that automated facial recognition could identify micro-expressions associated with discomfort, which may not be readily observable to clinicians. This technological capacity reduces human error and supports continuous pain monitoring in non-verbal populations, such as intensive care patients. Their research prefigured the later development of platforms like ePAT, which build on these principles to integrate multimodal assessment domains.

While the majority of studies reported psychometric equivalence between digital and paper-based assessments, some limitations are notable. The research largely focused on the validation of tools rather than their long-term clinical impact or sensitivity to change over time. Moreover, while the statistical correlations between digital and traditional assessments are high, these metrics do not necessarily account for contextual factors influencing assessment quality, such as lighting conditions, patient movement, or device calibration. These limitations show the need for continued validation across diverse clinical settings and populations. Pu et al. [44] also note practical constraints for facial analytics (e.g., visibility and pose), reinforcing the need for ongoing validation across settings and for procedures that mitigate lighting, occlusion, and movement artefacts.

Jonassaint et al. [43] provided a contrasting but complementary perspective through their evaluation of Painimation, a digital tool that uses animated visualisations rather than numeric or verbal descriptors. While not directly compared with APS, their study demonstrated that digital modalities could enhance communication and reduce reliance on numerical scales, particularly for patients who struggle to quantify or verbalise pain. Participants with higher visual analogue scale (VAS) pain ratings selected more intense animations, indicating concurrent validity between digital visual expression and traditional self-report metrics. This evidence expands the understanding of validity beyond numeric correlation, illustrating how digital tools may broaden the modes through which pain is meaningfully expressed and assessed.

Taken together, the findings from these studies suggest that digital pain assessment tools such as ePAT (PainChek), Painimation, and other facial recognition platforms offer concurrent validity and reliability when compared with traditional paper-based methods. Their capacity to reduce subjectivity, automate data capture, and facilitate consistency across assessors positions them as credible alternatives or complements to established scales like APS. However, the evidence base remains predominantly quantitative and instrument-focused, with limited exploration of longitudinal use, patient outcomes, and integration into broader clinical decision-making. Nevertheless, within the scope of existing studies, the convergence of results across multiple research teams and patient groups indicates that digital pain assessment tools provide an accurate and reliable means of evaluating pain, particularly in populations where traditional self-report is limited or unfeasible. In particular, Pu et al. [44] help bridge scale-level validity and behavioral mechanisms by showing that specific facial expressions tracked by digital tools are meaningfully aligned with observed pain in dementia, further legitimizing these platforms as credible complements to clinician judgement.

#### 3.6.2. Theme 2: Comprehensive Pain Evaluation Across Contexts (Rest vs. Movement)

An essential component of effective pain assessment lies in evaluating pain both at rest and during movement to ensure a comprehensive understanding of the patient’s pain experience. This distinction enables clinicians to discern variations in pain intensity and character that may otherwise remain undetected when assessment occurs under static conditions alone. The reviewed studies consistently identified the assessment of pain at both rest and during movement as a critical factor in achieving an accurate and comprehensive evaluation. This distinction was viewed as essential to understanding the full experience of pain and its potential impact on function. Across the included studies, researchers recognised that evaluating pain in multiple contexts provides clinicians with richer and more nuanced information, supporting the development of appropriate and responsive pain management strategies. Atee et al. [39,40,41] and Hoti et al. [42] all demonstrated that pain assessments conducted during movement consistently produced higher scores than those taken at rest, indicating the importance of capturing both static and dynamic expressions of pain to guide effective clinical decision-making.

Atee et al. [39] employed a random effects regression model to explore whether differences in pain scores were influenced by timing, context, or method of testing. They reported that the timing of the assessment such as the time of day did not significantly affect the results, indicating that pain scores were consistent across testing intervals. However, a notable difference emerged between pain assessments conducted at rest and those undertaken during movement. Correlation analysis demonstrated a significant positive relationship between ePAT and APS scores at rest (r = 0.880, 95% CI 0.845–0.907) and during movement (r = 0.894, 95% CI 0.855–0.922). These findings confirmed that both tools detected higher pain scores during movement, aligning with clinical expectations that physical activity often exacerbates discomfort. The close correlation across both states validated the capacity of the digital tool to detect pain reliably in dynamic clinical contexts.

Atee et al. [41] corroborated these findings in a subsequent study by comparing ePAT and APS scores using random effects modelling. Their data demonstrated significantly higher mean scores for pain during movement (7.3 ± 3.7) than at rest (4.0 ± 2.2), with a highly significant *p*-value (<0.0001). This result reinforces the importance of dual-context assessments and confirms the digital tool’s sensitivity to physiological changes associated with movement-induced pain. The researchers also verified that this variance was consistent across repeated measures, suggesting that digital assessment tools maintain psychometric stability across changing activity levels. The ability to detect pain variations between rest and movement enhances the clinical utility of these digital platforms, providing practitioners with richer data for interpretation and care planning.

Similarly, Atee et al. [40] found positive correlations between APS and ePAT across both assessment contexts. Their results indicated that the correlation between the two tools was not significantly influenced by the patient’s state of rest or movement, suggesting that the digital tool performs consistently across different physical conditions. Both APS and ePAT recorded higher mean scores when patients were in motion (11.44 ± 3.54) compared to when they were at rest (8.33 ± 3.34). This finding not only supports the reliability of ePAT in capturing pain fluctuations but also highlights its clinical relevance in dynamic care settings where patients engage in activities such as transfers, ambulation, or physiotherapy.

Hoti et al. [42] reinforced these observations in their evaluation of ePAT, reporting that mean ePAT scores were significantly higher during movement (11.44 ± 3.54) than at rest (8.33 ± 3.34). The reproducibility of these values across studies shows the consistency of digital assessments in capturing pain variations across different physical states. Importantly, the alignment of ePAT and APS scores across conditions demonstrates concurrent validity between digital and traditional assessment methods. This reliability across rest and movement contexts establishes digital tools as credible alternatives to paper-based scales in detecting both static and activity-related pain.

Despite these limitations, the collective findings indicate that dual-context pain assessment is consistently captured by digital tools, highlighting an area for further evaluation in clinical practice. Assessing pain exclusively at rest may underestimate its severity and impact, particularly in older adults and those with limited ability to communicate. For patients with dementia or cognitive impairment, digital tools capable of recognising facial micro-expressions and behavioural cues during movement offer a distinct advantage. By capturing variations in pain expression as the patient moves, digital platforms provide data that reflect the lived experience of pain more accurately than static assessments alone. This capacity aligns with the principles of person-centred care, which emphasise understanding pain as a multidimensional, context-dependent experience rather than a fixed symptom.

A further benefit of assessing pain at both rest and movement lies in its implications for treatment planning. Higher pain scores during movement often signal functional pain that can hinder mobility, independence, and participation in rehabilitation. The consistent detection of such patterns by digital tools indicates their potential role in informing clinical decisions around analgesic timing, physiotherapy scheduling, and mobility support. The studies reviewed suggest that digital pain assessment tools can generate more nuanced data that enable clinicians to distinguish between background pain and activity-related exacerbations, thereby guiding more individualised interventions.

Another important observation across these studies is that the correlation between rest and movement assessments was not confounded by extraneous factors such as time of day, assessor variability, or environmental conditions [39]. This finding enhances confidence in the stability of digital assessments, supporting their use in various care environments, including long-term care facilities and acute hospital settings. The ability to produce consistent results across repeated measures is essential for monitoring pain trajectories and evaluating treatment efficacy over time.

While these findings are promising, it is also important to recognise the limitations inherent in the current evidence. Most studies relied on cross-sectional, or validation designs rather than longitudinal frameworks, limiting insights into how rest and movement assessments might predict outcomes or inform ongoing pain management. Furthermore, while statistical correlations were observed, the studies did not systematically examine how clinicians interpret or act upon differences between rest and movement scores in practice. This represents an important gap in the literature, as the ultimate value of dual-context assessment lies not only in its psychometric properties but also in its influence on clinical decision-making and patient outcomes.

The digital platforms reviewed demonstrate that pain intensity and expression vary between rest and movement and that these variations can be reliably captured using technology-enhanced methods. The capacity of digital tools to detect and quantify these differences in real time presents a significant advancement over traditional, paper-based scales, which often capture pain as a static, single-point measure.

In summary, the assessment of pain at both rest and during movement is critical for obtaining a complete understanding of the patient’s pain experience. The evidence reviewed demonstrates that digital pain assessment tools, particularly ePAT (PainChek), provide valid and reliable evaluations across both contexts, generating more detailed assessment data than static, single-context measures. They are sensitive to changes in pain expression associated with physical activity and produce results that correlate strongly with established instruments such as the APS. These findings suggest that digital assessments can enhance the comprehensiveness of pain evaluation by capturing variation between rest and movement, generating more detailed assessment data than static, single-context measures. However, evidence linking dual-context digital assessment to changes in clinical decision-making or patient outcomes remains limited.

#### 3.6.3. Theme 3: Usability and Integration into Clinical Practice

The usability and clinical integration of digital pain assessment tools represent a critical determinant of their successful adoption in healthcare practice. While digital platforms demonstrate psychometric reliability, their value in routine care depends largely on their accessibility, ease of use, and compatibility with clinical workflows. Across the studies included in the scoping review, the user-friendliness of digital tools for clinicians and patients was a recurrent theme, with emphasis on time efficiency, device adaptability, and the potential to enhance documentation and data management. However, the evidence also indicates that effective implementation requires adequate training and user engagement to ensure these tools deliver their intended benefits. Collectively, findings from Atee et al. [39,41], Hoti et al. [42], Butler et al. [48], Jonassaint et al. [43], Lucey et al. [46], and Pu et al. [45] highlight the importance of usability as a bridge between technological innovation and clinical practicality.

Atee et al. [39] described the ePAT (PainChek) platform as a point-of-care application that utilises facial recognition technology through mobile smart devices. Its structure includes 42 descriptor items organised across six domains, each representing a distinct dimension of pain. The design allows the assessor to capture data quickly and receive automated scoring outputs. By removing the need for manual computation, ePAT minimises opportunities for arithmetic or recording errors and provides an immediate overall pain score. This automation represents a considerable improvement over traditional paper-based scales such as the Abbey Pain Scale (APS), which require manual scoring and documentation. Furthermore, Atee et al. [41] and Hoti et al. [42] both emphasised that ePAT’s ability to run on tablets and smartphones enhances its portability and accessibility, making it particularly suitable for use in residential aged care settings where mobility and rapid response are crucial.

While the studies consistently highlight the operational advantages of mobile-based pain assessment, they also recognise that successful use of these technologies depends on adequate training. Atee et al. [39] noted that clinicians would require familiarisation with the ePAT application to ensure consistent use and interpretation. Training is necessary not only for operating the device but also for understanding the facial recognition outputs and integrating these findings into clinical judgment. Although digital tools automate certain aspects of pain evaluation, professional oversight remains essential, particularly when interpreting behavioural indicators or determining treatment responses. The need for training was also echoed by Pu et al. [45], who found that staff confidence and familiarity with the patient population significantly influenced perceptions of the PainChek app’s effectiveness.

Pu et al. [45] conducted interviews with nursing staff and families regarding their experiences with PainChek in clinical settings. Participants reported that the app was generally easy to use and valued its capacity for mobile deployment. Staff particularly appreciated its ability to capture and record pain responses efficiently, reducing the need for manual data entry and simplifying comparisons of pain scores over time. Families viewed the tool positively, noting its potential to improve communication between staff and residents and to overcome language barriers. Importantly, both groups recognised that the tool’s value was maximised when used by staff familiar with the individual being assessed. This finding highlights the importance of combining digital assessment outputs with personalised knowledge of the patient’s baseline behaviours and expressions.

Butler et al. [48] provided additional evidence of user acceptability through the evaluation of PainCas, a digital self-reporting platform designed to replace paper questionnaires. PainCas enabled patients to record their pain electronically, with data automatically integrated into electronic records. Patients described the system as easier to use and more conducive to discussing pain with clinicians. Nurses similarly reported that the electronic format streamlined documentation processes, eliminating the need to scan or manually enter data from paper forms. Butler’s findings demonstrate that usability benefits extend beyond convenience, as digital systems can enhance record-keeping accuracy, improve data availability for multidisciplinary teams, and support continuity of care.

Jonassaint et al. [43] explored usability from a patient perspective through their evaluation of Painimation, a tablet-based application that allows patients to communicate pain using animated visualisations rather than numbers or words. While their study focused on patient experience rather than clinician usability, it provides important insights into the adaptability of digital platforms. Most participants (87%) agreed or strongly agreed that Painimation was a useful way to describe pain, suggesting that visual, interactive formats may improve engagement and comprehension among diverse patient populations. However, the study did not assess healthcare professionals’ views, representing an area for future research to ensure that clinician usability complements patient experience.

Lucey et al. [46] examined how continuous digital monitoring could support clinicians in capturing and tracking pain expressions. Their research demonstrated that automated systems could record facial action units continuously over time, providing clinicians with longitudinal data that could be reviewed retrospectively. This functionality allows practitioners to identify trends in pain progression or response to treatment without requiring additional manual input. The authors argued that such systems could reduce the subjectivity of assessments and enhance the accuracy of pain monitoring in patients who cannot communicate verbally, such as those in intensive care. From a usability perspective, the capacity for automated, continuous recording reduces the need for repeated manual assessments, thereby supporting workflow efficiency.

Despite these clear advantages, the studies also highlight limitations and considerations related to integration into practice. For example, Atee et al. [39] and Pu et al. [45] both indicated that the introduction of digital pain assessment tools requires institutional support, adequate training, and ongoing evaluation to ensure sustainable implementation. The initial learning curve associated with new technologies can present challenges for staff who are less digitally literate or resistant to change. Moreover, while devices such as tablets and smartphones enhance accessibility, their effective use in clinical environments depends on reliable connectivity, secure data management, and compatibility with existing electronic health record systems.

Environmental and workflow factors must also be considered. Digital tools that rely on facial recognition may require adequate lighting and clear visibility of the patient’s face to function optimally. These conditions may not always be achievable in busy or resource-limited settings. Therefore, while the evidence supports the feasibility and time efficiency of digital pain assessment, these practical constraints should inform implementation strategies to ensure consistent use across different clinical contexts.

Overall, the reviewed evidence describes favourable usability characteristics of digital pain assessment tools within the studied settings, provided that implementation is accompanied by sufficient training and infrastructure support. The studies show that these platforms offer time-saving benefits, improve documentation accuracy, and integrate well into modern digital healthcare environments. They also highlight the potential to enhance patient engagement and communication, particularly when tools are used collaboratively by clinicians and patients. However, successful integration depends on maintaining a balance between automation and clinical expertise—ensuring that digital outputs complement, rather than replace, professional judgment.

In conclusion, the usability and integration of digital pain assessment tools into clinical practice are underpinned by their capacity for mobility, automation, and enhanced documentation. Evidence from the reviewed studies demonstrates that digital platforms such as ePAT (PainChek), PainCas, and Painimation are perceived as efficient and user-friendly, supporting both clinicians and patients in the pain assessment process. Nevertheless, effective use requires structured training, institutional support, and attention to contextual factors such as lighting, device reliability, and digital literacy. When implemented with these considerations, digital pain assessment tools have the potential to streamline clinical workflows, reduce administrative burden, and enhance the accuracy and timeliness of pain documentation thereby supporting more consistent documentation and assessment processes within the studied settings.

#### 3.6.4. Theme 4: Enabling Person-Centred Pain Management and Future Directions

The included studies suggest potential mechanisms by which digital tools may support aspects of person-centred pain assessment, particularly in populations with communication barriers. The studies included in the scoping review demonstrate that digital platforms can facilitate the detection and documentation of pain, with potential relevance for individualised assessment in populations with communication barriers. While the evidence base remains largely focused on psychometric validation and usability, there are indications that these tools may support specific aspects of person-centred pain assessment within the studied contexts. The following synthesis draws upon findings from Atee et al. [39,40,41], Babicova et al. [47], Hoti et al. [42], Butler et al. [48], Jonassaint et al. [43], Lucey et al. [46], and Pu et al. [45] to examine how digital pain assessment tools contribute to improved pain detection and the development of personalised care strategies.

Digital pain assessment tools are designed to translate complex behavioural and physiological signals into quantifiable data that can guide clinical decision-making. Atee et al. [39,40,41] and Hoti et al. [42] demonstrated that ePAT (PainChek) achieves this by employing facial recognition algorithms to detect facial action units (AUs) associated with pain expression, along with structured observation of behavioural domains such as voice, movement, activity, and body indicators. These domains allow clinicians to determine when and where pain responses occur and to differentiate between the presence, intensity, and potential triggers of pain. The categorisation of pain into levels—ranging from “no pain” to “severe pain” enables the development of structured and personalised management plans. By producing objective, domain-based scores, digital tools support clinicians in identifying patterns of pain expression that may otherwise go unnoticed, particularly in patients unable to verbalise discomfort. Pu et al. (2023) [45] strengthen this rationale by specifying the facial action units most closely tied to observational pain in dementia, thereby clarifying which non-verbal cues may warrant particular attention in person-centred assessment and care planning.

The reliability and sensitivity of these tools in detecting pain are crucial for enabling more individualised interventions. Atee et al. [40] reported that ePAT’s validation against the Abbey Pain Scale confirmed its suitability for assessing pain in individuals with reduced self-report capacity, such as residents with dementia. This finding is relevant to clinical assessment in populations with limited self-report capacity, as digital tools may assist in identifying pain where communication barriers are present. By providing a reliable and reproducible method for pain detection, digital tools enhance clinicians’ confidence in identifying pain accurately, thereby facilitating appropriate analgesic administration and care planning.

Hoti et al. [42] similarly demonstrated the clinometric properties of ePAT, affirming its potential to improve assessment accuracy for residents who cannot verbally communicate. The capability of digital tools to record and analyse subtle behavioural and facial cues provides clinicians with an additional layer of evidence upon which to base clinical judgments. This advancement moves beyond traditional observational methods, which can be influenced by assessor subjectivity or fatigue, offering a more objective approach to identifying distress. The inclusion of automated scoring and documentation features further supports continuity of care, allowing pain assessments to be shared and reviewed across multidisciplinary teams.

Butler et al. [48] explored the clinical implications of digital pain documentation through the PainCas system, which digitised self-assessment and integrated results into electronic records. Nurses reported that the use of PainCas improved the quality and consistency of documentation, with significant increases in recorded pain-relevant topics as confirmed through chi-square testing. Patients also found the educational and informational aspects of the tool beneficial, with 64.7% reporting that it enhanced their understanding of pain and its management. From a person-centred perspective, these findings suggest that digital tools can empower patients by facilitating greater engagement in their own care and by promoting shared understanding between patients and clinicians. Furthermore, 72.2% of nurses indicated that PainCas enhanced their ability to document and review pain assessments efficiently, supporting a more responsive approach to pain management.

Jonassaint et al. [43] provided a different but complementary perspective on person-centred care through the development of Painimation, an animated, visual platform that allows patients to communicate pain using imagery rather than numbers or words. This approach acknowledges that pain is a subjective, multifaceted experience that may not be fully captured through conventional numerical rating scales. The study found that patients with higher pain intensity on the Visual Analogue Scale (VAS) tended to select more intense animations, indicating good concurrent validity between visual and numerical representations of pain. Importantly, participants reported that the animations allowed them to express pain more accurately and intuitively, reducing the linguistic and cognitive barriers associated with standard scales. This innovation highlights how digital tools can enhance inclusivity in pain communication and support more individualised assessment approaches, aligning with the core principles of person-centred care.

Lucey et al. [46] extended this concept by demonstrating how automated facial recognition systems can provide continuous monitoring of pain expressions over time. This continuous data output enables clinicians to observe changes in pain intensity or frequency, offering a longitudinal perspective that traditional one-off assessments cannot provide. By tracking pain trends, clinicians can evaluate the effectiveness of interventions and adjust treatment plans accordingly. Such longitudinal monitoring is particularly valuable in critical care and dementia care settings, where patients’ ability to communicate fluctuates and where behavioural cues may be subtle or transient. Although Lucey et al. (2011) [46] acknowledged limitations in patients with facial registration difficulties or uncontrolled head motion, their findings illustrate the potential of digital tools to generate ongoing, objective evidence to support personalised care planning.

Pu et al. [45] further illuminated how digital pain tools influence perceptions of care among healthcare staff, patients, and families. Through qualitative interviews, they found that staff viewed PainChek as a valuable alternative for assessing pain in residents who cannot verbalise discomfort, particularly in culturally and linguistically diverse populations. Families expressed optimism about the tool’s ability to facilitate communication and ensure that their relatives’ pain was recognised and addressed promptly. Staff emphasised that the tool’s effectiveness was enhanced when assessors possessed an in-depth understanding of the patient’s usual behaviours and expressions. These finding highlights that while digital tools provide valuable data, their person-centred impact depends on the combination of technological precision and human familiarity with the patient. When viewed alongside Pu et al. [44] the qualitative reports of acceptability and communication benefits are anchored by empirical evidence that the facial signals captured by the app map onto observed pain behaviors. This convergence experiential acceptability plus signal validity supports the person-centred case for digital tools in populations with limited verbal communication.

While the reviewed studies collectively indicate that digital pain assessment tools enhance the detection and understanding of pain, they also reveal a significant research gap concerning their direct impact on clinical outcomes. Most studies focused on validating the accuracy and usability of digital platforms rather than evaluating their influence on pain management decisions, care planning, or patient outcomes. As a result, there is limited evidence on how the integration of these tools translates into measurable improvements in pain control, functional recovery, or quality of life. Future research should therefore move beyond validation studies to include longitudinal designs that examine how digital assessments inform care pathways, analgesic use, and patient-reported outcomes over time. A practical next step, informed by Pu et al. [44] is to evaluate whether care pathways that explicitly monitor high-informative facial cues (e.g., brow lowering, horizontal mouth stretch) lead to timelier analgesic adjustments, reduced untreated pain episodes, and improved functional outcomes in dementia care.

Another key consideration for future implementation relates to staff training and clinical integration. Both Atee et al. [39] and Pu et al. [45] noted that the effectiveness of digital tools depends on clinicians’ ability to interpret and act upon the generated data. Training programmes should therefore focus not only on technical competence but also on developing interpretative skills and understanding the clinical implications of digital pain metrics. Ensuring that digital assessments complement, rather than replace, clinical judgment will be vital for maintaining the human dimension of person-centred care.

In conclusion, the current body of evidence indicates that digital pain assessment tools hold considerable promise for advancing person-centred pain management. By enabling objective detection of pain through facial recognition, behavioural analysis, and interactive visualisation, these tools provide clinicians with comprehensive data to guide tailored interventions. They enhance communication between patients, families, and healthcare providers, support accurate documentation, and facilitate continuity of care. However, the translation of these benefits into demonstrable patient outcomes remains an area requiring further empirical exploration. As healthcare systems continue to integrate digital technologies, future research should focus on evaluating the long-term clinical impacts of digital pain assessment tools, their role in improving care quality, and their potential to reshape pain management for adults and older adults in diverse clinical settings. When implemented thoughtfully, digital pain assessment tools can serve as catalysts for truly person-centred and responsive pain care.

These themes reflect findings from a small and methodologically heterogeneous body of literature, primarily comprising validation and feasibility studies conducted in high-income, institutional care settings.

## 4. Discussion

This scoping review synthesised evidence from ten studies examining digital pain-assessment tools in adults and older adults and identified four key themes: the comparative validity and reliability of digital tools, the comprehensive evaluation of pain at rest and during movement, the usability and integration of these platforms into clinical practice, and their contribution to person-centred pain management. The findings indicate that digital assessment tools consistently show high correlation with established methods such as the Abbey Pain Scale (APS) and perform well across different patient states [39,40,41,42,47]. They also capture differential pain levels at rest versus during movement [39,40,41], demonstrate practical usability for clinicians and patients [45,48], and show promise in enabling tailored interventions for non-verbal and cognitively impaired patients [40,47]. Placing these findings in the wider context of pain assessment and digital health highlights both their significance and the remaining evidence gaps. This review makes a novel contribution by synthesising evidence on both the psychometric performance and the practical, person-centred application of digital pain-assessment tools in adults and older adults. Unlike previous reviews, our analysis highlights the implications of real-world clinical integration and identifies critical gaps that must be addressed to ensure equitable and effective adoption of digital assessment technologies.

Although this review was framed around adults and older adults, the included evidence primarily reflects older adults with dementia in residential aged-care settings. Consequently, the findings are most directly applicable to this population and context. Evidence relating to other adult groups, individuals with non-dementia cognitive impairment, community-dwelling populations, or settings with differing resource constraints remains limited. These boundaries should be considered when interpreting the broader applicability of digital pain-assessment tools.

It is important to interpret these themes in light of heterogeneity across the included studies. Although several studies reported favourable psychometric and usability outcomes, findings varied according to tool modality, target population, and context of use. Facial-recognition-based tools evaluated in dementia-focused residential care settings demonstrated more consistent performance than self-report platforms or tools assessed in broader adult populations. In addition, many studies were limited by small sample sizes, context-specific validation, and limited reporting of blinding or follow-up, which may contribute to variability in reported outcomes and restrict confidence in generalisability.

The reported psychometric performance of the digital tools aligns with a growing body of literature suggesting that automated, facial-recognition and behavioural codification methods may enhance objectivity in pain assessment, particularly within dementia-focused care settings [50,51,52]. Traditional self-report measures are limited by communication difficulty, cognitive impairment, and cultural or emotional influences [53,54], making observational and digital tools particularly relevant for older adults with dementia or reduced capacity to self-report. Evidence from broader studies shows that observational tools like the Critical Care Pain Observation Tool (CPOT) achieve high sensitivity in non-verbal populations [55,56]. The convergence of findings highlights conceptual alignment between digital assessment approaches and broader calls to improve pain detection in populations with limited communication. This trajectory reflects conceptual alignment with international policy goals, NICE NG97 [14] emphasises appropriate pain assessment methods for people living with dementia, and the WHO Global Strategy on Digital Health [57] promotes evidence-based digital innovation that improves diagnostic accuracy and safety for vulnerable populations.

A further conceptual limitation within the evidence base is the predominant reliance on the Abbey Pain Scale (APS) as the reference standard for validating digital tools. Although APS is frequently used in dementia care, it is subject to observer subjectivity and demonstrates variable reliability across settings. Consequently, high correlation with APS, although indicative of concurrent validity should not be interpreted as definitive evidence of diagnostic accuracy. Future validation studies should incorporate multi-modal comparison standards, including self-report where feasible, independent clinician assessments, and alternative observational tools, to reduce dependence on a single benchmark and strengthen the robustness of digital tool evaluation.

Moreover, the theme of comprehensive assessment capturing pain both at rest and during movement reflects an evolution in pain evaluation that recognises the dynamic nature of pain. As the studies in the review show, pain scores are higher during movement, emphasising the need for assessment beyond static conditions [39,40,41,42]. In the broader literature, older adults face variable pain experiences tied to mobility, activity, and functional status [58,59,60]. Thus, digital tools that maintain validity across contexts offer a more nuanced view of pain, supporting interventions targeted at functional limitation [61,62]. NICE NG193 (Chronic pain in over-16s) [63] explicitly calls for comprehensive, person-centred assessment that explores both rest and movement, supporting this multidimensional approach. Integrating these tools within NICE-aligned frameworks may support more consistent pain evaluation; however evidence linking their use to improved functional outcomes remains limited.

Usability and clinical integration emerge as critical for realising the potential of digital tools. The literature on digital health for pain management emphasises that clinician adoption depends not just on tool accuracy but on workflow compatibility, training, data management, and user engagement [64]. The studies in the review echo this: while ePAT and related apps are designed for mobile deployment and automated scoring [39,41,42], successful implementation in practice also requires staff familiarity and institutional support [44]. In a nursing context where time pressure and documentation burden are significant [60,65], digital platforms that offer rapid, standardised assessments and integrate with electronic health records may reduce assessment variability and support consistent pain monitoring. The WHO digital-health framework [57] highlights that such integration must be accompanied by training, governance, and interoperability planning to ensure safe and sustainable uptake across healthcare systems.

The final theme enabling person-centred pain management connects digital assessment to broader goals of tailored and responsive care. The ability of digital platforms to categorise pain levels, detect subtle behavioural cues, and generate longitudinal data [46] places them in line with contemporary calls for personalised health technologies that support decision-making and care planning. The broader digital health literature emphasises how artificial intelligence and automation can contribute to precision medicine and patient engagement [66,67,68,69]. In older adults and those with cognitive impairment who cannot reliably self-report, digital tools may support assessment processes used in nurse-led decision-making and facilitate shared understanding among patients, families, and clinicians [60,70,71,72]. This aligns with NICE guidance [14,64] advocating co-produced care planning and with WHO [57] principles of equity, accessibility, and safety in digital innovation. However, as the review has shown, evidence remains limited on how these tools translate into improved outcomes such as analgesic use, functional recovery, or quality of life.

It is important to note, however, that the evidence supporting person-centred benefits remains largely inferential. Most included studies focused on tool validity, usability, or observational concordance rather than patient outcomes, symptom trajectories, or the impact on care quality. As such, claims regarding enhanced person-centred care reflect the theoretical potential of digital tools rather than empirically demonstrated effects. Nevertheless, digital assessment outputs could feasibly support clinical decision-making by, for example, prompting analgesic review when scores escalate, guiding the timing of interventions such as movement-based care or physiotherapy, or enabling longitudinal monitoring to evaluate treatment response. Future research should prioritise evaluating these direct clinical impacts to confirm whether digital assessment meaningfully improves person-centred pain management.

Despite the encouraging findings, key limitations within the evidence base must be addressed. Most studies are validation or feasibility designs with relatively small samples and limited longitudinal follow-up. The broader literature similarly highlights that digital health interventions must be evaluated for effectiveness, cost–benefit, and implementation outcomes, not merely accuracy [73]. Further, while digital tools promise objectivity, underlying algorithmic bias, data quality, and ethical considerations such as patient privacy and algorithmic transparency remain underexplored. For older adults with dementia or complex health needs, the interplay of facial morphology, movement artefact, and algorithmic sensitivity may challenge accuracy an issue currently under-reported in the literature. Finally, although the review studies demonstrate comparability to APS, they tend to evaluate tool performance in controlled or pilot settings rather than in routine, resource-constrained clinical environments. This limits our understanding of real-world effectiveness, scalability, and sustainability, issues central to the WHO digital-health strategy’s call for evaluation of long-term clinical impact and health-system readiness.

Beyond methodological limitations, the use of AI-enabled pain-assessment tools raises important algorithmic and ethical considerations. Facial-recognition systems may inadvertently encode biases related to age, sex, or ethnicity, which could affect detection accuracy across diverse populations [74]. These tools also require the capture and storage of sensitive biometric data, presenting privacy and consent challenges, particularly for older adults or individuals with cognitive impairment who may have diminished capacity to provide informed consent [75]. Furthermore, the lack of transparency surrounding proprietary algorithms limits clinicians’ ability to understand how pain scores are generated or to challenge questionable outputs. As digital pain-assessment tools expand in clinical use, issues of algorithmic fairness, privacy protection, data governance, and explainability will be critical for ensuring equitable and trustworthy implementation.

### 4.1. Recommendations

Based on the synthesis of evidence, several recommendations for clinical practice, research, and education can be proposed. Based on the mapped evidence, digital pain-assessment tools warrant further evaluation in clinical settings where communication barriers are prominent, particularly in dementia care. Implementation should be accompanied by structured training programes that focus on both technical proficiency and interpretive competence, ensuring that clinicians understand how to apply digital assessment results in clinical decision-making. From a research perspective, there is a need for longitudinal and outcome-focused studies to evaluate how digital pain assessments influence analgesic prescribing, patient-reported outcomes, and care quality. Mixed-methods approaches could also provide valuable insights into user experiences, barriers to adoption, and contextual factors influencing effectiveness. In educational contexts, nursing and allied health curricula should include content on digital literacy and the clinical application of digital assessment tools to prepare future practitioners for technology-enabled care environments. Finally, policy frameworks should support standardization and quality assurance in digital pain assessment, ensuring interoperability and data security across healthcare systems. Any practice-oriented implications drawn from this review should be interpreted as provisional and hypothesis-generating, reflecting the limited number of studies, their narrow clinical contexts, and the predominance of early-stage validation designs.

### 4.2. Strengths and Limitations

This scoping review provides a comprehensive synthesis of current evidence on digital pain assessment in adults and older adults, bringing together studies that examine psychometric validity, clinical usability, and person-centred outcomes. A key strength is the inclusion of a range of study designs that collectively demonstrate both the quantitative reliability and qualitative acceptability of digital assessment tools. The review also highlights the convergence of evidence across different research teams, strengthening the credibility of the findings. However, several limitations should be acknowledged. The review is limited to ten studies, many of which involve small sample sizes and pilot or validation designs, restricting generalizability. Most studies focus on tool development and validation rather than evaluating clinical outcomes or implementation success. Furthermore, heterogeneity in study methodologies, populations, and digital platforms makes direct comparison challenging. Finally, as the review only included studies available in English, relevant evidence published in other languages may have been excluded. Despite these limitations, the findings provide an important foundation for understanding how digital pain assessment tools can enhance accuracy, usability, and person-centred care, while highlighting key priorities for future research and practice. The heterogeneity of study designs and outcomes, combined with recurring methodological limitations, limits direct comparison across tools and reinforces the exploratory nature of the conclusions.

## 5. Conclusions

This scoping review indicates that the available evidence for digital pain-assessment tools relates to facial-recognition-based technologies evaluated in dementia care within high-income residential aged-care settings. While these preliminary findings suggest acceptable psychometric performance and usability in these contexts, the overall evidence base remains small, context-specific, and dominated by validation studies. As such, conclusions regarding broader adult populations, non-dementia cognitive impairment, community or acute care settings, and low-resource environments remain largely aspirational. Future research should prioritise independent evaluations across more diverse populations and settings, as well as studies examining implementation and clinical impact.

Despite these strengths, the review highlights significant gaps: the preponderance of small-scale validation studies, the paucity of data linking tool use to clinical outcomes, and limited exploration of cost-effectiveness, implementation barriers, and equity impacts. To move the field forward, future research must adopt robust longitudinal designs, evaluate the real-world effects on care delivery and patient experience, and examine how organisational, educational and ethical frameworks influence uptake.

In conclusion, while digital pain-assessment tools represent a promising advancement in measurement and care, their true value will depend on thoughtful evaluation within clinical practice, supported by rigorous evidence, clinician engagement, and governance safeguards.

## Figures and Tables

**Table 1:** Study Characteristics.

Author	Year	Country	Type of Digital Pain Assessment	Participants	Sample Size	Study Design	Setting	Aim	Key Findings
Atee et al. [39]	2017	Australia	ePAT	Residents with dementia	*n* = 34	quantitative	Care home	To describe a new pain assessment tool, ePAT that integrates technologies to benefit patients with cognitive impairment and to evaluate its psychometric properties compared to the Abbey Pain Scale	The ePAT demonstrated excellent concurrent validity and good discriminant validity. Inter-rater reliability score was good overall, while internal consistency was excellent. ePAT has psychometric properties which make it suitable for use in non-communicative patients with dementia. ePAT also has the advantage of automated facial expression assessment which provides objective and reproducible evidence of the presence of pain
Atee et al. [40]	2017	Australia	ePAT	60-year-old + aged care home residents	*n* = 40	quantitative	Care home	To investigate the psychometric properties of ePAT	The ePAT is a suitable tool for the assessment of pain in individuals with moderate to severe Dementia
Atee et al. [41]	2018	Australia	ePAT	63–84-year-old residents with dementia	*n* = 10	quantitative	Dementia specific aged care facility	To examine the interrater reliability of the electronic pain assessment Tool (ePAT)	ePAT demonstrates good reliability properties, which supports its appropriate use in residents with advanced dementia
Hoti et al. [42]	2018	Australia	ePAT	Dementia patients over 65	*n* = 34	quantitative	Residential aged care facilities	Examine clinimetric properties (clinical utility and predictive validity) of the ePAT	The clinimetric properties demonstrated were excellent, thus supporting the clinical usefulness of the ePAT
Jonassaint et al. [43]	2018	USA	Painimation	Pain patients	*n* = 170	Quantitative	Pain medicine clinic	To develop and test Painimation. This study examines the utility of abstract animations as a measure of pain.	Using animations may be a faster and more patient-centred method for assessing pain and is not limited by age, literacy level, or language; however, more data are needed to assess the validity of this approach. To establish the validity of using abstract animations (“painimations”) for communicating and assessing pain, apps and other digital tools using painimations will need to be tested longitudinally across a larger pain population and also within specific, more homogenous pain conditions.
Pu et al. [44]	2023	Australia	PainChek	Residents 65 years and over	*n* = 46	Secondary data analysis as part of randomised control trial—quantitative	Aged care facility	To identify specific facial expressions associated with pain behaviours using the PainChek	Six specific facial expressions were associated with observational pain scores in residents with dementia. Results indicate that automated real-time facial analysis is a promising approach to assessing pain in people with dementia
Pu et al. [45]	2023	Australia	PainChek & PARO	Residents and carers and relatives	*n* = 13	Interviews Qualitative Randomised control trial	Residential aged care facility	Experiences of residents with dementia, family, and formal carers in relation to pain assessment and management for residents with dementia, the use of the PainChek app for pain assessment, and the use of a social robot PARO for pain management in residents with dementia.	PainChek and PARO, is promising to improve pain assessment and reduce pain for people with dementia. Barriers to using technology include limited staff training and the implementation of person-centred care
Lucey et al. [46]	2011	USA	AAM	Patients with shoulder injuries		Quantitative		We show that the AAM can deal with these movements and can achieve significant improvements in both AU and pain detection performance compared to the current-state-of-the-art approaches which utilize similaritynormalized appearance features only.	To evidence that AAM can be used to detect pain
Babicova et al. [47]	2021	UK	Painchek	Residents of care home	*n* = 22	quantitative	Care home	The aim of this study was to further validate PainChek^®^, with a population living with dementia in a UK care home	PainChek^®^ has demonstrated to be a valid and reliable instrument to assess the presence and severity of pain in people with moderate-to-severe dementia living in aged care
Butler et al. [48]	2016	USA	PainCas	Hospital patients	*n* = 147	mixed methods	Hospital	To determine the impact of PainCAS on documentation of pain and opioid risk evaluations and outcomes	use of the PainCAS assessment improves documentation of chart elements in clinical notes and is associated with increased discussion of key, pain relevant topics during the clinical visit.

## Data Availability

No new data were created or analyzed in this study.

## Supplementary Materials

The following supporting information can be downloaded at: https://www.mdpi.com/article/10.3390/healthcare14020149/s1, Supplementary File S1. PRISMA ScR Checklist; Supplementary File S2. Search Strategies; Supplementary File S3. Summary of reported quantitative outcomes.

## Abbreviations

The following abbreviations are used in this manuscript:

AI	Artificial Intelligence
APS	Abbey Pain Scale
AAM	Active Appearance Model
AU	Action Unit (s) (Facial movement components in facial recognition analysis)
COPT	Critical Care Pain Observation Tool
ePAT	Electronic Pain Assessment Tool
ICU	Intensive Care Unit
JBI	Joanna Briggs Institute
NHS	National Health Service
NICE	National Institute for Health and Care Excellence
NMC	The Nursing and Midwifery Council
OSF	Open Science Framework
PEO	Population, Exposure, Outcome
PRISMA-ScR	Preferred Reporting Items for Systematic Reviews and Meta-Analyses extension for Scoping Reviews
PRN	Pro re nata (Latin: “as needed”)
WHO	World Health Organization
