# Supplementary material for: Digital Approaches to Pain Assessment Across Older Adults: A Scoping Review
Source: Healthcare (Basel). 2026 Jan 7;14(2):149. doi: 10.3390/healthcare14020149 (PMC12841086; doi:10.3390/healthcare14020149)
Supplement: Supplementary file 1 [file healthcare-14-00149-s001.zip › Supplementary file 2- search terms.pdf]

Supplementary File – Search Terms

PsycINFO:

TX("digital pain assessment" OR "electronic pain assessment" OR "electronic pain measurement" OR Painimation OR PainChek)	**AND**	TX(Elderly OR "older adults" OR geriatric OR "geriatric patients" OR elders OR "older person" OR "older people" OR "older patients" OR "senior citizens" OR "frail elderly" OR adult OR "middle aged" OR aged)

PubMed:

("Pain Measurement"[MeSH]<br>OR "Pain Assessment"[MeSH]<br>OR "digital pain assessment"[Title/Abstract]<br>OR "electronic pain assessment"[Title/Abstract]<br>OR "electronic pain measurement"[Title/Abstract]<br>OR Painimation[Title/Abstract]<br>OR PainChek[Title/Abstract])	**AND**	("Aged"[MeSH]<br>OR "Adult"[MeSH]<br>OR "Middle Aged"[MeSH]<br>OR elderly[Title/Abstract]<br>OR "older adults"[Title/Abstract]<br>OR geriatric[Title/Abstract]<br>OR "geriatric patients"[Title/Abstract]<br>OR elders[Title/Abstract]<br>OR "older person"[Title/Abstract]<br>OR "older people"[Title/Abstract]<br>OR "older patients"[Title/Abstract]<br>OR "senior citizens"[Title/Abstract]<br>OR "frail elderly"[Title/Abstract]<br>OR adult[Title/Abstract]<br>OR "middle aged"[Title/Abstract]<br>OR aged[Title/Abstract])

CINAHL (All):

(MH "Pain Measurement+"<br>OR MH "Pain Assessment+"<br>OR "digital pain assessment"<br>OR "electronic pain assessment"<br>OR "electronic pain measurement"<br>OR Painimation<br>OR PainChek)	**AND**	(MH "Aged+"<br>OR MH "Adult+"<br>OR MH "Middle Aged+"<br>OR elderly<br>OR "older adults"<br>OR geriatric<br>OR "geriatric patients"<br>OR elders<br>OR "older person"<br>OR "older people"<br>OR "older patients"<br>OR "senior citizens"<br>OR "frail elderly"

		OR adult<br>OR "middle aged"<br>OR aged)

MEDLINE:

(exp Pain Measurement/<br>OR exp Pain Assessment/<br>OR "digital pain assessment".ti,ab.<br>OR "electronic pain assessment".ti,ab.<br>OR "electronic pain measurement".ti,ab.<br>OR Painimation.ti,ab.<br>OR PainChek.ti,ab.)	**AND**	(exp Aged/<br>OR exp Adult/<br>OR exp Middle Aged/<br>OR elderly.ti,ab.<br>OR "older adults".ti,ab.<br>OR geriatric.ti,ab.<br>OR "geriatric patients".ti,ab.<br>OR elders.ti,ab.<br>OR "older person".ti,ab.<br>OR "older people".ti,ab.<br>OR "older patients".ti,ab.<br>OR "senior citizens".ti,ab.<br>OR "frail elderly".ti,ab.<br>OR adult.ti,ab.<br>OR "middle aged".ti,ab.<br>OR aged.ti,ab.)
