# Supplementary material for: Digital Approaches to Pain Assessment Across Older Adults: A Scoping Review
Source: Healthcare (Basel). 2026 Jan 7;14(2):149. doi: 10.3390/healthcare14020149 (PMC12841086; doi:10.3390/healthcare14020149)
Supplement: Supplementary file 1 [file healthcare-14-00149-s001.zip › Supplementary File 3 Table.pdf]

Supplementary File 3: Quantitative outcomes reported across included digital pain-assessment studies

Study	Tool	Study focus	Outcome type	Metric(s) reported	Key quantitative findings	Population / Setting
**Atee et al., 2017**	ePAT	Psychometric validation	Concurrent validity; inter-rater reliability; internal consistency	r, κ, α	r = 0.882 vs Abbey Pain Scale; κ = 0.74; α = 0.925	Dementia; residential aged care
**Atee et al., 2018**	ePAT (PainChek®)	Psychometric validation	Inter-rater reliability; discriminative validity	CCC, κ, κw	CCC = 0.92; κ = 1.00 (rest), 0.59 (movement); κw ≈ 0.69–0.72	Dementia; residential aged care
**Atee et al., 2017**	ePAT (Electronic Pain Assessment Tool)	Psychometric validation	Concurrent validity; discriminant validity; predictive validity; reliability	Pearson's r; weighted κ; ICC; Cronbach's α	Concurrent validity vs APS: r = 0.911 (overall; rest r = 0.896; movement r = 0.904); inter-rater reliability: κw = 0.857 (95% CI 0.819–0.895); ICC = 0.904 (95% CI 0.885–0.921); internal consistency α = 0.950; higher pain scores with movement vs rest ($p < 0.0001$)	Residents with moderate–to-severe dementia in residential aged care (34 residents; 400 paired assessments)
**Hoti et al., 2018**	ePAT (PainChek®)	Clinimetric accuracy	Diagnostic accuracy; clinical utility	Sensitivity, specificity, AUC, CUI	Sensitivity 96.1%; specificity 91.4%; AUC = 0.98; CUI+ = 0.936	Dementia; residential aged care
**Babicova et al., 2021**	PainChek®	Psychometric validation	Concurrent validity; reliability	r, κ, ICC, α	r = 0.818 vs APS; κ = 0.719; ICC = 0.68; α = 0.81	Dementia; UK care homes
**Butler et al., 2016**	PainCAS	Implementation / process	Documentation outcomes	χ^2, mixed models	Improved documentation ($p < .001$); no pain outcome differences	Adults; primary care
**Jonassaint et al., 2018**	Painimation	Feasibility / construct exploration	Associations; discrimination	r, AUC	r = 0.35 with PainDETECT; AUC = 0.59	Adults; chronic pain

**Lucey et al., 2011**	Facial AU model	Algorithm performance	Classification accuracy	A′ (ROC)	A′ = 84.7 for pain vs no-pain detection	Experimental dataset
**Pu et al., 2024**	PainChek®	Associational modelling	Regression associations	β coefficients	AU20 $\beta = 1.67$; AU4 $\beta = 0.76$ (all $p < .05$)	Dementia; residential aged care
**Pu et al., 2023**	PainChek® + PARO	Implementation	Feasibility / acceptability	Descriptive	High acceptability; no psychometric metrics	Dementia; residential aged care

Notes: r = correlation coefficient; κ = Cohen's kappa; κw = weighted kappa; ICC = intraclass correlation coefficient; AUC/A′ = area under ROC curve; CUI = clinical utility index.
